# Contributions of national and global health estimates to monitoring health-related Sustainable Development Goals in Thailand

**DOI:** 10.3402/gha.v9.32443

**Published:** 2017-05-22

**Authors:** Kanitta Bundhamcharoen, Supon Limwattananon, Khanitta Kusreesakul, Viroj Tangcharoensathien

**Affiliations:** a International Health Policy Program, Ministry of Public Health, Thailand; b Faculty of Pharmaceutical Science, Khon Kaen University and International Health Policy Program

**Keywords:** National health estimates, global health estimates, burden of diseases, Sustainable Development Goals, Thailand

## Abstract

The Millennium Development Goals (MDGs) triggered increased demand for data on child and maternal mortality for monitoring progress. With the advent of the Sustainable Development Goals (SDGs) and growing evidence of an epidemiological transition towards non-communicable diseases, policy makers need data on mortality and disease trends and distribution to inform effective policies and support monitoring progress. Where there are limited capacities to produce national health estimates (NHEs), global health estimates (GHEs) can fill gaps for global monitoring and comparisons. This paper draws lessons learned from Thailand’s burden of disease study (BOD) on capacity development for NHEs, and discusses the contributions and limitation of GHEs in informing policies at country level. Through training and technical support by external partners, capacities are gradually strengthened and institutionalized to enable regular updates of BOD at national and sub-national levels. Initially, the quality of cause of death reporting in the death certificates was inadequate, especially for deaths occurring in the community. Verbal autopsies were conducted, using domestic resources, to determine probable causes of deaths occurring in the community. This helped improve the estimation of years of life lost. Since the achievement of universal health coverage in 2002, the quality of clinical data on morbidities has also considerably improved. There are significant discrepancies between the 2010 Global Burden of Diseases (GBD) estimates for Thailand and the 1999 nationally generated BOD, especially for years of life lost due to HIV/AIDS, and the ranking of priority diseases. National ownership of NHEs and effective interfaces between researchers and decision makers contribute to enhanced country policy responses, while sub-national data are intended to be used by various sub-national-level partners. Though GHEs contribute to benchmarking country achievement compared with global health commitments, they may hamper development of NHE capacities. GHEs should encourage and support countries to improve their data systems and develop a data infrastructure that supports the production of empirical data needed to underpin estimation efforts.

## Background

Since the Millennium Development Goals (MDGs) were adopted at the UN Millennium Summit in 2000, governments, international institutions, and donors have demanded accurate data for monitoring MDG progress [1]. However, lack of quality data in low- and middle-income countries is widely acknowledged as an ongoing challenge. Accurate data contribute to identifying health gaps and priorities for health investment in relation to global targets [2]. To facilitate tracking of health systems’ progress over time and comparisons across countries, international statistics experts have produced ‘global health estimates’ (GHEs) despite encountering country data shortfalls [3].

At the conclusion of the MDG era in 2015, countries adopted the Sustainable Development Goals (SDGs). These are far more ambitious and extensive in scope than the MDGs and place significant demands on countries to monitor progress towards the SDG targets regularly until 2030 [4].

In the light of the ongoing inadequacies in the availability and quality of country data, GHEs will continue to be needed for monitoring progress towards SDGs. Yet GHEs cannot replace the need for improved data infrastructure and capacities in countries where there is a lack of data or poor data quality. As the production of GHEs continues to expand, the challenge is to assess the extent to which a GHE can be used to improve the availability and quality of data and support policy decisions at country level.

This study aims to draw lessons from experience in capacity development of ‘national health estimates’ (NHEs) through the burden of disease (BOD) program in Thailand, and to discuss the contributions and limitations of GHEs in informing policies at country level.

## Experiences of NHEs: Thai BOD

### The policy demands for a national BOD in Thailand

Since the World Development Report 1993 ‘Investing in Health’ [5] was published, Thai policy makers have become more familiar with the concept of Disability-Adjusted Life Years (DALYs), a summary measure of fatal and non-fatal health outcomes in a population. There is a growing consensus on the potential value of such measures for policy and planning [6–9]. Interest in BOD was also stimulated by consultations among member states on health system performance assessment, for which BOD is one of the critical components [10]. The WHO has invested in a series of technical workshops providing training and capacity-building for member states on the development of BOD.

The first study on BOD in Thailand was completed in 1997 [11], supported by the Health Systems Research Institute. The study has limitations regarding quality of data, which were mainly derived from the mortality and morbidity database at the Health Statistics Division of the Ministry of Public Health (MOPH).

BOD requires exhaustive data, including data related to mortality, incidence, prevalence of all diseases in a population, the age at onset, duration, and disease progression. Thailand has a quite well-designed health information system (HIS) [12]. There are two types of HIS data, i.e. population-based and health facility-based data-sets. Population-based data sources comprise the civil registry, population census, and household surveys regularly conducted by the National Statistical Office (NSO). Facility-based data cover health service records, the disease notification surveillance system, and certain disease registries.

Mortality statistics are essential for the estimate of BOD, in particular years of life lost (YLL) and related causes. Problems arose when it became apparent that more than 40% of reported deaths were ascribed to ‘ill-defined’ causes or ‘garbage codes’ as referred to by the Global Burden of Disease (GBD) report [13]. The MOPH recognized the need to improve the quality of cause-of-death (COD) ascertainment. In response, it convened the first national verbal autopsy (VA) study to determine COD for those who died in 2000 without proper medical certification. The initial plan was to improve the accuracy of COD information in the routine death registration system by introducing a system of medical certification for deaths occurring at home in parallel with regular VA studies to enable adjustment of the routine data. However, this plan has not been progressing well due to the complexity of coordination in the field as well as scant policy and funding support from the MOPH. In practice, the results from the VA study were used to verify and re-estimate COD in the 1999 Thai BOD estimation, followed by the 2004 BOD report [14].

The BOD uses a variety of sources of morbidity data, which are collected by different authorities both within and outside the MOPH. Continued effective inter-sectoral collaboration among related agencies, and private sector providers, is required.

### Assessment of data quality for BOD

Since the BOD estimates both fatal and non-fatal health outcomes in a population, it is essential to understand the potential measurement bias from various sources of data. The first step of BOD estimation is to assess the quality of data in terms of completeness and accuracy (Table 1).

**Table 1.** Quality assessment of data.

Data sources	Health indicator	Quality assessment	
		Coverage	Accuracy
Vital registration	Age-sex-specific mortality	Overall 95% completeness of all death events, less completed among perinatal, neonatal, and the oldest age group so that death events are not registered	NA
Vital registration	Cause-specific mortality	Same as above	Up to 40% ill-defined cause, in particular where deaths take place at home
Hospital data	Mortality	35% of total deaths took place in hospitals	Still 20% ill-defined cause
	Incidence and prevalence of disease	NA	Quality control process
Disease notification	Incidence, mortality, case fatality	In principle it covers all events.	Suspected and confirmedcases are reported;it was used for diseasesurveillance and outbreakcontrol purposes
		Public health personnel have a duty to notify their local authority of suspected cases of certain infectious diseases.	
		Often public non-MOPH and private hospitals do not comply with notification of diseases to the MOPH	
Household health survey	Incidence, prevalence of selected diseases	Large household survey is a national representative sample	Recall bias and laypeople report

Note: NA: not applicable.

The civil registration system, which records almost all deaths, is the main source of mortality statistics in Thailand and is thus the key contributor to the accuracy of YLL estimates. An assessment supported by the University of Queensland and the WHO in 2012 rated the Thai registration system as ‘satisfactory,’ requiring minor adjustments in an otherwise well-functioning system [15]. The survey of population change, conducted every decade (inter-census survey) by the NSO, reported 2% and 5% incompleteness of mortality registration in 2005–6 and 1995–6 respectively [16,17]. However, higher levels of incompleteness were found in other studies [18,19], e.g. at 8.69% (95% confidence interval [CI] 8.65–8.72%) by a dual records system estimation [18]. Use of indirect demographic methods found higher levels of incompleteness in mortality registration [20]. Inaccurate COD assignment for each mortality event is an unresolved problem, as 49% of deaths during 1950–2000 were coded as ill-defined [13].

The disease notification surveillance system has regularly produced the Weekly Epidemiological Surveillance Report (WESR) since the 1970s by the MOPH Bureau of Epidemiology [21]. The WESR covers all major communicable diseases such as HIV/AIDS, diarrhea, and tuberculosis (TB). It is intended to provide timely information on disease outbreaks for control and response purposes and publishes data on notifiable disease patterns and trends nationwide. Its strengths are the accuracy of disease case reporting, timeliness, and rapid response. The time-series data are useful for trends analysis of epidemics. However, the system has important limitations. In particular, there is incomplete coverage of surveillance and reporting of notifiable diseases due to the fact that most of the public non-MOPH and private hospitals and clinics do not adhere to reporting standards, despite the fact that a significant proportion of services for urban populations are provided by this sector.

The Universal Coverage Scheme (UCS) introduced in 2002 requires the payment system to include individual service utilization data [22,23]. This has resulted in significantly improved accuracy of clinical diagnosis in institution-based services. In addition, there has been considerable improvement in data infrastructure, including hardware, software, and regular capacity-building and auditing. A data-auditing system helps ensure accuracy of the data. The National Health Security Office (NHSO) collects data on services rendered to beneficiaries covered by the UCS, more than 75% of the total population.

Population-based surveys are an indispensable source of data on population coverage, complementing the institutional coverage rates estimated from routine clinical data collection. The NSO conducts nationally representative household surveys on a regular basis, in particular Health and Welfare Surveys (HWS). The National Health Examination Survey (NHES), conducted every five years, contributes significantly to data generation for specific disease conditions. However, surveys based on interviews have limitations due to recall bias and lack of specificity and sensitivity for specific disease diagnoses. The NHES, which includes clinical/biomedical measurements, provides more accurate diagnoses though at a significantly higher cost.

### Capacity development and institutionalization of NHEs

Following the publication of the first GBD estimates in 1997 [24], the WHO convened a series of training workshops bringing together researchers and health managers from several member states. The workshops were useful in capacity-building for Thai researchers and health managers. In 2000, the Thai MOPH conducted the first estimation of the Thai BOD, establishing a BOD working group with joint financial support from the WHO Country Office, and setting up the first national VA study [25]. The work was undertaken with technical support from academic institutions in Australia and financial support from AUSAID. The first BOD report in Thailand was published in 2002 which attracted the attention of the MOPH’s high-level executives and relevant stakeholders [26]. Recognizing its usefulness, policy makers endorsed continued BOD estimates.

The success of the first study brought about a new funding opportunity from the Wellcome Trust and the creation of a project call ‘Setting Priority using Information on Cost-Effectiveness’ (SPICE) [27]. SPICE is a joint collaboration between a group of Thai researchers and the University of Queensland. The project included a capacity-building program, awarding Master’s and doctoral fellowships from the University of Queensland. Importantly, the second national VA study was undertaken under the auspices of this project [27] and the findings were applied to the 2004 national BOD study.

The institutionalization of the national BOD was gradually achieved, largely with funding support from the ThaiHealth Foundation. Today the BOD is a major program within the International Health Policy Program (IHPP) which is internationally well known due to its engagement in health system and policy research (HSPR). The BOD program contributed significantly to a number of health promotion research programs, e.g. the Centre for Alcohol Study (CAS), Food and Health Promotion Policy, and Non-communicable, Chronic Disease (NCD) Networking Program; these programs were developed with support from the ThaiHealth Foundation. Evidence from the BOD also supports further research work in these research programs.

In addition to the BOD estimates at national level, further work includes sub-national analysis [28,29]. This is particularly useful for situation analysis and benchmarking across 77 provinces and 13 public health regions. In 2009, the total DALYs lost for the population of 63.4 million in the whole country was 10.2 million. Region1, a cluster of 8 upper northern provinces, had the highest DALYs rate, and the lowest was in the Bangkok metropolitan Region; these ranged from 34% higher to 24% lower than the national DALYs rate. It also reveals different patterns of disease burden across the health regions. While this is useful for policy making at health region level, the quality of COD and the completeness of morbidity data have yet to be strengthened.

### Quality improvement of mortality data

The quality of COD is unsatisfactory because the majority of deaths occurred at home and were reported by laypersons [30]. Deaths with underlying causes coded to ‘garbage codes’ [13] were high at 53% in 1999 (59% of total home deaths, 34% of total hospital deaths). Following the first VA in 1999, a number of actions have been undertaken by the MOPH. It conducted several rounds of training for health personnel working in hospitals and health centers. An intervention that aimed to have all home deaths certified by medical personnel was not successful partly due to limited staff capacity. Subsequently, a shortened VA was introduced for health center personnel to interview the deceased’s relatives before getting a death registered. However, this was not well received as it lengthened the process of death registration. Later on, registrars were trained to be aware of the accuracy of COD and use a shortened VA to improve it. However, there is considerable variation in how this is implemented in practice.

### Policy utilities of NHEs

The first BOD estimates greatly raised awareness among policy makers in the MOPH by generating evidence on HIV/AIDS as the leading cause of premature death in 1999, followed by road traffic injuries and stroke [26]. The vital registration system reported only 6400 deaths due to HIV/AIDS, compared with the BOD estimate of 51,100. This was the first national estimation of mortality due to HIV/AIDS, apart from the ASEAN Epidemic Model (AEM) [31]. The BOD exercise not only generated new data but also presented the information in a way that was readily interpretable from a policy perspective. For example, by contrast to routine health statistics reports, the BOD data were offered in diverse formats, such as leading causes of death and DALYs by age and gender and rankings of the proportions of deaths by cause. The BOD exercise also adjusted COD distributions obtained from the routine vital registration by VA studies. These factors rendered the BOD estimates more compelling for policy makers.

The 1999 BOD rankings of causes of deaths and disabilities were cited in the vast majority of health policy and strategic plan documents that followed, such as the five-year national socioeconomic development plan, the MOPH strategic plan, and other statistical references [32–37]. Based on the estimates of attributable risk fractions generated by the BOD exercise, public health priorities were increasingly directed towards tobacco control, alcohol abuse, prevention of road traffic injuries, and drug addiction. During the period 2001–2005, several health reforms were undertaken simultaneously, requiring BOD evidence. Among these were the establishment of the ThaiHealth Foundation in 2001, and the NHSO in 2002.

The evidence from the 1999 Thai BOD report, showing the shifting disease burden to non-communicable diseases (NCDs), injuries, and mental health disorders, threw a spotlight on the need for effective health promotion interventions [38]. From its origins in tobacco control, the ThaiHealth Foundation was established by law as a public organization, with its own legal status and governing body, chaired by the Prime Minister with funding from a 2% surcharge on alcohol and tobacco excise tax. The establishment of the ThaiHealth Foundation has enabled innovative health promotion programs with various stakeholders outside the MOPH and supports effective inter-sectoral actions.

Thailand is increasingly adopting an evidence-based policy strategy in health, requiring solid documentation of the size of a problem and the ability to monitor and evaluate intervention effectiveness. For example, evidence from the BOD provides the foundation for the MOPH strategic plan and its various departments including Disease Control; the ThaiHealth strategic plan; key performance indicators of the NHSO; and the national NCD and risk control plan. Furthermore, based on evidence from the BOD, Health-Adjusted Life Expectancy (HALE) was set as a target in the 10-year strategic plan for the ThaiHealth Foundation and the MOPH [32,35].

**Figure 1.**
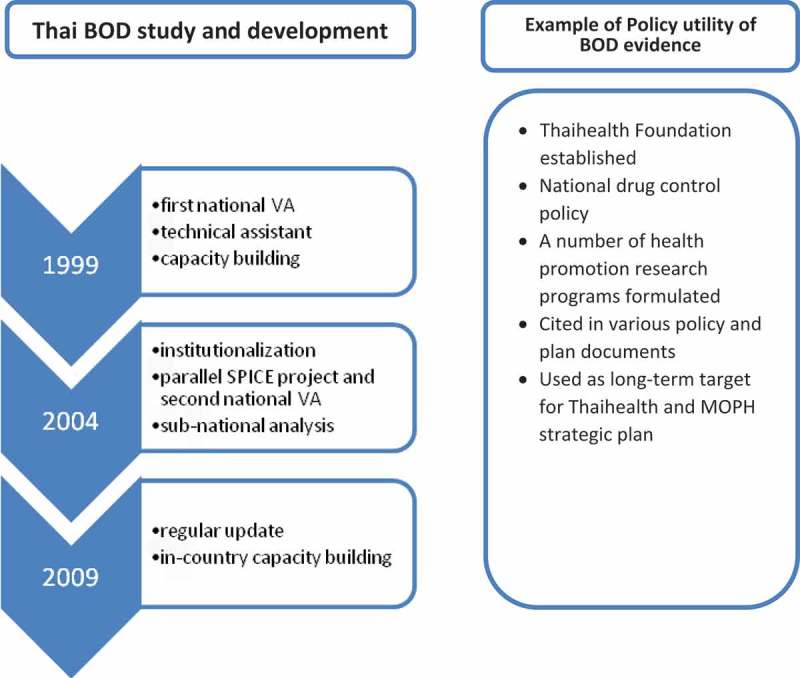
Summary of Thailand’s BOD development.

Although the meaure of DALYs lost was cited as a major priority-setting criterion for health interventions in many MOPH policy and planning documents, it is not considered feasible to use as an outcome indicator because it is, by its nature, a summary measure and based on complex and time-consuming methods. Instead, the MOPH uses routine statistics, in particular mortality and morbidity, as monitoring indicators because they are collected routinely, comparable over time, and are disaggregated by administrative level. Figure 1 summarizes the three phases of BOD development from inception to institutionalization, and its contributions to policy uses.

### Discrepancies between GHEs and NHEs

The World Health Reports 1999–2004 include DALY estimates globally and by WHO region [39–44]. The 2010 GBD reports estimates of deaths and DALYs by cause for individual countries. Since then the GHE publication has been produced on an annual basis [6]. The GBD estimates are useful for comparing and benchmarking across countries. However, the BOD profiles estimated by the GBD are quite different from the Thai estimates with respect to both methodology and findings.

First, the Thai BOD used 2009 as its reference year, while GBD used the data-set for 2010. Second, the 2010 GBD adopted a more sophisticated estimation approach than the Thai BOD, which used the recommended methods provided by the 1990 GBD. The 2010 GBD used modified methods including a new reference standard life table and age classification, no age weights, no discount rate for YLLs (formerly 3% per annum was used), a prevalence non-fatal burden approach, and more detailed classification of disease sequelae [45]. All these modifications resulted in divergent estimates of priority health priorities from the two sources.

Table 2 compares the estimates of YLLs from the 2009 Thai BOD and the 2010 GBD reports [46]. The GBD revealed that HIV/AIDS was the leading cause of YLLs while the Thai BOD reported road traffic injuries as the leading cause of DALYs, with HIV/AIDS ranked fifth. In 2015, meetings between the BOD program and GBD teams generated much discussion on the inaccuracy of the GBD estimates, particularly on HIV/AIDS. Furthermore, while the GBD presented data on the change of disease burden patterns between 1990 and 2010, it did not capture the reduction of HIV/AIDS deaths due to universal coverage of ART since 2005. Evidence shows that high coverage of universal ART improved quality of life among people living with HIV/AIDS and drastically reduced HIV/AIDS-related mortality [47–49]. Some general conclusions emerged from the discussions. One was that the increasing complexity of the GBD approach was not readily replicable within countries. Another was that the GBD statistical models tended to have considerable inertia so that recent changes in country health indicators were not fully captured. In fact, it was noted that the underlying data upon which the GBD models were developed did not reflect all available empirical data in the country. Conflicting results from GBD and country disease profiles can create confusion among policy makers, as was the case in Thailand.

**Table 2.** Top 20 causes of YLLs; comparing GBD and Thai BOD estimates.

	Thai BOD (2009)*			GBD (2010)		
	Disease	YLLs (x 1000)	%	Disease	YLLs (x 1000)	%
1	Traffic accidents	1108	11.1	HIV/AIDS	1064	8.5
2	HIV/AIDS	764	7.7	Ischaemic heart disease	971	7.8
3	Stroke	753	7.6	Traffic accidents	867	7.0
4	Ischaemic heart disease	555	5.6	Lower respiratory tract infections	796	6.4
5	Liver cancer	537	5.4	Stroke	787	6.3
6	Diabetes	485	4.9	Liver cancer	631	5.1
7	Cirrhosis	335	3.4	Suicides	456	3.7
8	Bronchus & Lung cancer	268	2.7	Bronchus & Lung cancer	395	3.2
9	Homicide and violence	231	2.3	Diabetes	380	3.0
10	Suicides	231	2.3	Homicide and violence	406	3.3
11	Drownings	224	2.2	Cirrhosis	341	2.7
12	COPD	216	2.2	Drownings	321	2.6
13	Nephritis & nephrosis	215	2.2	Chronic kidney disease	291	2.3
14	Lower respiratory tract infections	186	1.9	COPD	269	2.2
15	Tuberculosis	182	1.8	Tuberculosis	242	1.9
16	Low birth weight	160	1.6	Preterm birth complications	182	1.5
17	Birth trauma & asphyxia	151	1.5	Congenital anomalies	175	1.4
18	Colon & rectum cancer	143	1.4	Other cardio & circulatory	170	1.4
19	Cervix uteri cancer	142	1.4	Colorectal cancer	162	1.3
20	Falls	139	1.4	Typhoid fevers	206	1.6

Notes: *YLLs without age weight and discounting. COPD: .

### Feedback and verification loop

A commonly expressed concern at country level was the lack of adequate consultation with countries for verification, feedback, and reality checking. The GBD, based on statistical modelling and incorporating multiple assumptions, often introduced changes to the methodologies in response to scientific inputs but without giving any consideration to the impact such changes would have on policy relevance and utility of the results to countries. The absence of consultation, verification, and reality checks with country partners was a factor in inhibiting the use of the GBD estimates by country experts and decision makers. More recently, the WHO and IHME have offered more opportunities for country feedback which is greatly welcomed.

### Inconsistent estimates over time

Another source of confusion can occur when new update estimates become available and a new set of estimations is produced. Such an example is the trends in maternal mortality estimates developed by the WHO, UNICEF, UNFPA, World Bank, and UN Population Division [50–52]. In the case of Thailand, the latest estimate of annual change between 1990 and 2013 was −2% while that between 1990 and 2010 was −0.6%. Although it was stated that the latest estimates should not be compared to or extrapolated from previously published estimates, it would be rather problematic to use new estimates every time they were produced.

### Strengths and limitations of GHEs and NHEs

Table 3 compares strengths and limitations of GHEs and NHEs. The GBD produces comparable statistics across countries using the same methods of estimation and thus facilitates country benchmarking and monitoring progress. GHEs bring together strong technical capacities and are well funded. GHEs, however, do not capture sub-national-level information, which is much needed by policy makers for specific policy interventions. The availability of GHEs may hamper countries’ commitment to develop capacities to produce NHEs.

**Table 3.** Strengths and limitations of GHEs and NHEs.

	NHEs	GHEs
Strengths	Traceability of cause of and ability to identify sub-national health gaps	Comparability
	Sustainability	Benchmarking
	Country ownership and policy consumption	Standard robustness of estimates
		Well-funded programs
Limitations	Lack of capacity to generate NHEs	Difficult to identify causes and gaps at sub-national level
	Comparability with other country NHEs	May hamper commitment to strengthen capacity to generate NHEs

NHEs, using different methods, are not designed for benchmarking across countries nor to support global tracking. The ownership of NHEs and sub-national estimates contribute to specific policy interventions. Policy demand for NHEs supports sustainability and quality improvement. The major limitation is lack of institutional capacities to develop and sustain NHEs in most low- and middle-income countries.

## Lessons learned

### Use of NHEs for policy decision, monitoring and evaluation, and reprogramming

The DALY, a summary health measure at the population level, is useful for priority setting in policy formulation and for monitoring health outcomes in policy implementation. The production and use of DALYs on a global policy platform raise awareness and attention among national policy makers. GHEs play an important role in stimulating Thai policy makers to have accurate data for decision making.

The knowledge and understanding of the estimates among policy-makers and relevant technocrats through series of meetings and workshops facilitated the use of national BOD estimates and generated the culture of using evidence for policy making. Furthermore, interactive platforms relevant to country contexts and a sense of ownership of the national estimates have proved far more useful in supporting the use of DALYs in policy formulation than the GHEs generated from outside agencies without interaction with country partners.

### Relevance of GHEs to country reality

While GHEs attempt to provide accurate health measurement across the globe, they may overlook some country-specific issues. The 1990 GBD did not single out certain diseases that are of relevance in many countries. For example, leptospirosis was prevalent among Thai farmers but not included in the GBD’s disease lists. Likewise, thalassemia, a genetic disease prevalent in Thailand, was not covered by the GBD. With country capacities to estimate and the availability of data, both diseases were fully covered in the 1999 Thai BOD estimates.

The number of YLLs from HIV/AIDS in the 2010 GBD is very different from the 1999 national BOD estimates. As previously noted, the GBD estimates fail to reflect the significant mortality reductions following the inclusion of ART in the universal coverage program in 2005.

### Institutional capacity-building

Strengthening research capacity in low- and middle-income countries is one of the prerequisites for achieving development goals [53]. The experience of Thailand has shown that country capacity-building and institutionalization of NHEs are the key for improving accuracy of country data, maximizing their use in decision making, and thus contributing to the estimates’ purpose for health improvement.

Building and sustaining the institutional capacity of country agencies to generate and use NHEs is the cutting edge in prioritizing policies. Imbalanced investments by donor agencies favoring GHEs result in lost opportunities to strengthen country capacities to estimate and use evidence for priority setting, and create dependency on GHEs which may not always correctly reflect country realities.

Whereas global attention to GHEs may trigger countries’ awareness of the need for better data, those promoting GHEs have yet to move the agenda beyond ‘in-house estimates and modeling’ to ‘out-of-house empowering’ the country partners, and work closely with them to verify and check the reality.

### Unfinished agenda: quality of COD

Given that both NHEs and GHEs rely heavily on death data from civil registration systems, it is vital that countries improve the completeness and quality of death registration and ascertainment of COD at both health facility and community levels. The Health Metrics Network (HMN) guidelines on self-assessment of Civil Registration and Vital Statistics (CRVS) [54] offered an initial step in this direction but much more needs to be done. Country ownership of national health estimates is a prerequisite for policy use. Recent regional and global initiatives will increase policy makers’ awareness of their obligations to strengthen CRVS [55].

In Thailand, despite extensive coverage of health services and local civil registration offices, little progress has been made on improving the quality of COD ascertainment. The MOPH unit responsible has undertaken a number of measures and initiatives as described earlier. Unfortunately, the COD has not been perceived as a priority in the national health development plans; the unit has received limited financial support and has limited staff. Despite the lack of active support for implementation in order to improve COD in the Vital Registration systems, the training of registrars and local health workers in the use of VA for out-of-hospital deaths resulted in increased awareness of the needs for accurate COD ascertainment. Thus, between 1999 when the first VA study was conducted and 2014, the proportion of ill-defined codes of COD decreased from 53% to 31% (45% of total home deaths, 13% of total hospital deaths).

Monitoring health-related SDGs requires representative and comparable estimates. This essentially relies on the quality of local data, which is often limited in developing countries. A key challenge is premature mortality from non-communicable diseases, an indicator that requires good quality of COD data. Mortality from non-communicable diseases usually occurs in older age groups and from our data it is often misclassified as pneumonia and septicemia. While the data accuracy is improving over time, such mortality-reporting data can create problems in monitoring progress towards SDGs.

### Prioritizing actions over measurement

While setting and achieving health targets is essential, there is more work to do with identifying effective interventions to meet the targets. The latter need commitments, time, and programmatic effort and should not be superseded by how best and accurately to quantify key indicators. While the national average of health outcomes masks health inequities at sub-national level, there is a need to identify measurable indicators at sub-national level to which the NHEs can contribute.

### The way forward

This paper points to the importance of national health estimates in informing policy. A few synergistic actions are required to strengthen capacities for the advancement of NHEs. For example, improve data platforms, in particular the CRVS and population-based health surveys by national statistics agencies, for monitoring achievement of SDGs; improve capacity in data analysis and translating data into policy messages; and improve capacity to link together evidence and use it for policy making.

The development of national health estimates requires consultative meetings with local stakeholders, including clinicians, epidemiologists, data authorities, and disease control program officers. This gives opportunities for data feedback, leading to data improvement. As measurement and analytical skills in low- and middle-income countries are limited, collaboration with academic institutions and support from global health and development partners are essential.

## Conclusion

Our study has shown that accurate NHEs contribute not only to policy utility in a country, but also to GHEs. GHEs may benefit global actors and development partners, by providing a benchmark across countries on the achievements of SDGs. They may trigger countries to improve their data systems, strengthen data analysis, and rigorously validate empirically reported data. It is essential, however, that a country should have a functioning HIS that supports the production of empirical data. While development partner resources are often directed towards the generation of representative health estimates required for planning and monitoring, equal attention should be given to strengthening country capacity for national and sub-national health estimates. Generating health estimates that do not link with accountability would be less useful and a waste of resources.

## Paper context

Monitoring country progress on health-related Sustainable Development Goals requires accurate cause of death data. Lack of data from low- and middle-income countries poses the need for global health estimates developed by international agencies. This paper presents lessons learned from Thailand, drawing an example of burden of disease to shed light on the need for capacity-building at country level for valid measurement. Concerted efforts and partnership are needed between national and global health estimates.

## Terminology and acronyms

GBD – Global Burden of Disease calculations developed for all countries using a standard statistical methodology; conducted by international agencies such as the World Health Organization (WHO) and academic institutions such as the Institution for Health Metrics and Evaluation (IHME) at the University of Washington.

NBD – National Burden of Disease calculations for specific countries based on methods and approaches developed for GBD but using empirical country data and assumptions.

GHEs – Global health estimates for specific indicators developed by United Nations (UN) agencies (UN.AIDS – Joint United Nations Programme on HIV/AIDS, UNICEF – United Nations International Children's Emergency Fund, UNFPA – United Nations Population Fund, UN Population Division, World Bank, WHO) or by the IHME.

NHEs – National estimates for specific indicators using country-based empirical data and national statistical imputations and assumptions. The NHEs feed into the development of country burden of disease studies.

BOD – Burden of Disease calculations summarizing the overall impact of premature mortality and disability on the overall health status of a population by major cause groups.

DALYs – Disability Adjusted Life Years, a summary measure of years of healthy life lost due to fatal and non-fatal health outcomes in a population.

YLLs – Years of Life Lost due to premature mortality.

YLDs – Years of life living with a disability.
